# Prognostic value of quality of life and functional status in patients with heart failure: a systematic review and meta-analysis

**DOI:** 10.1186/s43044-024-00532-z

**Published:** 2024-08-05

**Authors:** Abraish Ali, Asad Ali Siddiqui, Izza Shahid, Harriette G. C. Van Spall, Stephen J. Greene, Marat Fudim, Muhammad Shahzeb Khan

**Affiliations:** 1 Department of Medicine, Dow University of Health Sciences, Baba-e-Urdu Road, Karachi, 74200 Pakistan; 2 Division of Preventive Cardiology, Houston Methodist Academic Institute, Houston, TX USA; 3 Department of Medicine and Department of Health Research Methods, Evidence, and Impact, McMaster University, Hamilton, ON Canada; 4 Research Institute of St Joe’s, Hamilton, ON Canada; 5 Duke Clinical Research Institute, Duke University School of Medicine, Durham, NC USA; 6 Division of Cardiology, Duke University School of Medicine, Durham, NC USA; 7 Department of Medicine, Duke University School of Medicine, Durham, NC USA

**Keywords:** Functional health status, Prognosis, Mortality, Hospitalization

## Abstract

**Background:**

Functional health status is increasingly being recognized as a viable endpoint in heart failure (HF) trials. We sought to assess its prognostic impact and relationship with traditional clinical outcomes in patients with HF.

**Methods:**

MEDLINE and Cochrane central were searched up to January 2021 for post hoc analyses of trials or observational studies that assessed independent association between baseline health/functional status, and mortality and hospitalization in patients with HF across the range of left ventricular ejection fractions to evaluate the prognostic ability of NYHA class [II, III, IV], KCCQ, MLHFQ, and 6MWD. Hazard ratios (HR) with 95% confidence intervals were pooled.

**Results:**

Twenty-two studies were included. Relative to NYHA I, NYHA class II (HR 1.54 [1.16–2.04]; *p* < 0.01), NYHA class III (HR 2.08 [1.57–2.77]; *p* < 0.01), and NYHA class IV (HR 2.53 [1.25–5.12]; *p* = 0.01) were independently associated with increased risk of mortality. 6MWD (per 10 m) was associated with decreased mortality (HR 0.98 [0.98–0.99]; *p* < 0.01). A 5-point increase in KCCQ-OSS (HR 0.94 [0.91–0.96]; *p* < 0.01) was associated with decreased mortality. A high MLHFQ score (> 45) was significantly associated with increased mortality (HR 1.30 [1.14–1.47]; *p* < 0.01). NHYA class, 6MWD (per 10 m), KCCQ-OSS, and MLHFQ all significantly associated with all-cause mortality in patients with HF.

**Conclusion:**

Identifying such patients with poor health status using functional health assessment can offer a complementary assessment of disease burden and trajectory which carries a strong prognostic value.

**Supplementary Information:**

The online version contains supplementary material available at 10.1186/s43044-024-00532-z.

## Background

Assessing functional health capacity is an important and recommended component of heart failure (HF) care [1]. Indeed, when acknowledging the potential debilitating effects of HF on physical, social, and psychological health status, many patients may value improved quality of life more than prolonging survival [2, 3]. Cardiovascular societies such as the American Heart Association (AHA) [4] and the European Society of Cardiology (ESC) [5] as well as patient advocate groups and regulatory bodies, such as the Food and Drug Administration (FDA) [6], actively advocate for the inclusion of patient-reported outcomes (PRO) as an endpoint complementary to mortality, cardiovascular events, and hospitalization in HF patients. Functional health assessment, which is often evaluated by the use of health-related quality of life (HRQoL) tools, enables an accurate surveillance of disease burden [4] due to their ability to assess common HF symptoms which are otherwise infrequently reported, such as fatigue and anxiety, thereby assisting clinicians in making informed clinical decisions. However, due to the complex nature of HF care and limited time and resources, the assessment of functional health status in HF patients is often sidelined in routine practice and infrequently included within primary outcomes of HF randomized trials [7]. Clarifying the relationship between traditional clinical outcomes such as mortality and hospitalization and their association with various functional status endpoints may further highlight the importance of these quality-of-life scores. Moreover, the degree to which functional status outcomes yield similar prognostic value in HFrEF and HFpEF is unclear, with prior studies demonstrating inconsistent results [8, 9]. Likewise, based on recent analyses, functional health assessment can vary considerably across racial/ethnic groups [10, 11] and geographic regions [12]. In this context, we aimed to conduct a systematic review and meta-analysis to comprehensively evaluate the prognostic ability of New York Heart Association (NYHA), 6-minute walk distance (6MWD), Kansas City Cardiomyopathy Questionnaire (KCCQ), and Minnesota Living with Heart Failure Questionnaire (MLHFQ) with the clinical endpoints of mortality and hospitalization in patients with HF. In addition, we examined regional differences with respect to tools for health or functional status.

## Methods

This meta-analysis follows the Preferred Reporting Items for Systematic review and Meta-Analyses (PRISMA) and AHA guidelines for systematic reviews and meta-analyses [13, 14]. The MEDLINE and Cochrane central databases were searched up to January 2021. No time or language restrictions were set. Table S1 outlines the search strategy. All articles retrieved were transferred to Endnote Reference Library (Version X8.1; Clarivate Analytics, Philadelphia, Pennsylvania) software, where duplicates were identified and removed. Titles and abstracts of all articles were independently screened by two reviewers (AAS and AA). Full texts of all shortlisted articles were then read to confirm relevance. To ensure no relevant articles were missed, bibliographies of all relevant studies and review articles were also screened. Any discrepancies between the two reviewers were resolved by consulting a third reviewer (IS).

Studies were included if they fulfilled the following eligibility criteria: (a) included patients with any HF; (b) reported all-cause mortality and/or hospitalization in HF patients; (c) reported disease-specific functional assessment tools rather than generic tools when predicting mortality and/or hospitalization; and (d) reported the following cutoff values: (≥ 5 for KCCQ, > 45 for MLHFQ and ≤ 200 m or 10 m increments for 6MWD) and classes [II, III, and IV] for NYHA. (e) Studies that were post hoc analyses of trials or observational studies that assessed independent association between baseline health/functional status and mortality and/or hospitalization.

Two reviewers (AAS and AA) independently extracted data pertaining study characteristics and baseline characteristics such as the number of participants, publication year, HF classification (classified according to reduced or preserved ejection fraction), left ventricular ejection fraction (LVEF), geographic location, length of follow-ups, and mean/median ages. Geographical regions were dependent on the location of where each study was coordinated. This was divided into 4 distinct locations, namely North America (including Canada and US), Europe (including any country in Europe), multi-regional (including countries from North American and Europe), and rest of the world.

The risk of bias was assessed using the Newcastle–Ottawa quality assessment scale for observational studies (Table S2) [15] and the Cochrane risk of bias assessment tool for RCTs (Figure S1) [16]. The Newcastle–Ottawa scale consists of three domains: (a) selection; (b) comparability; and (c) outcome. The quality was appraised using numbers such that the maximum score was 9, and a study with 7–9 had low risk, 4–6 had high risk, and 0–3 had very high risk of bias. The Cochrane tool had seven domains, namely random sequence generation, allocation concealment, blinding of participants and personnel, blinding of outcome assessment, incomplete outcome data, selective outcome reporting, and other biases [16]. In addition, we used the Gill and Feinstein criteria [17] to review how well quality of life was being measured in the publications by assessing how well they dealt with conceptual issues and methodology, with each criteria having several components as presented in Table S3. Any discrepancies between the 2 reviewers were resolved by consulting a third reviewer (IS). Publication bias assessment could not be conducted as the number of studies within each data set were less than 10.

Outcomes of interest included mortality and hospitalization using the following predictor variables: NYHA functional classes (I, II, III, IV) 6MWD test (≤ 200 m or 10 m increments), KCCQ (≥ 5 point increase in overall summary score [OSS]), and MLHFQ (> 45 score). Behlouli et al. [18] define a MLHFQ score > 45 representing poor QOL with a validation accuracy of 91%. Scores above this threshold indicate a significant burden of heart failure symptoms for KCCQ, an improvement or worsening of ≥ 5 points and indicate a minimal clinically important difference in health status [19, 20]**.** A 6MWD < 200 m could identify patients with stable HF who are at markedly increased risk of death [21]. This cutoff point, chosen using Akaike’s information criterion, also resulted in the highest Harrell’s concordance index (c‐index) [22]. The threshold of 10 m increments in 6MWD was included based on its availability in the pooled studies.

RevMan (version 5.3; Copenhagen: The Nordic Cochrane Centre, The Cochrane Collaboration, 2014) was used for all statistical analyses. The results were presented as hazard ratios (HR) with 95% confidence intervals (CI), which were pooled using a generic invariance weighted random effects model. Forest plots were created to visually assess the pooled analysis. A geographic regional subgroup analysis was also conducted for each functional health assessment tool where possible. Leave-one-out sensitivity analysis was conducted to see if any one study disproportionately affected the results. Heterogeneity across studies was evaluated using Higgins I^2^, where a value less than 50% was considered acceptable and a value > 75% was considered significant. A p value < 0.05 was considered significant in all cases.

## Results

**Fig. 1:**
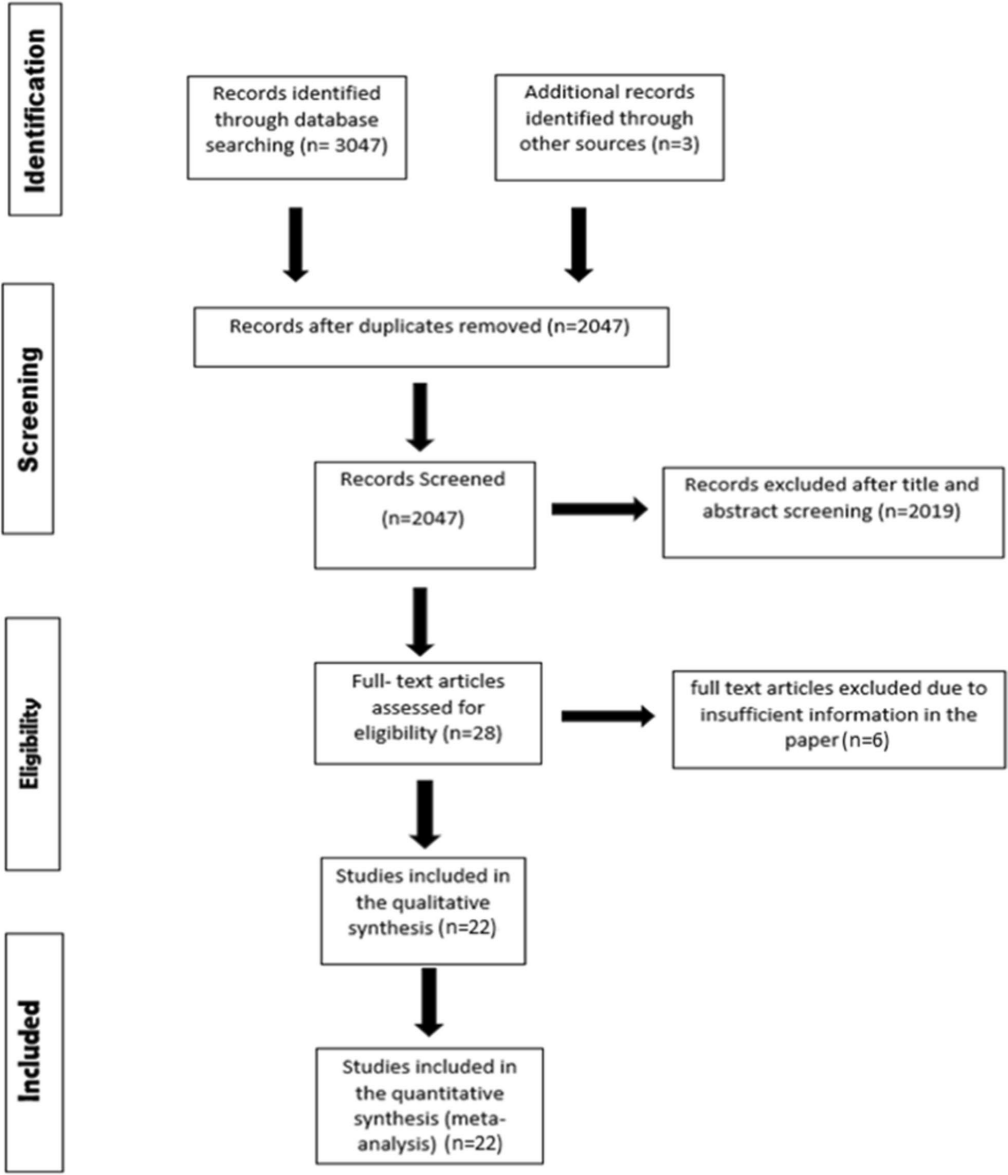
PRISMA flowchart outlining literature search. [CHF, Coronary Heart Failure; EF, Ejection Fraction; IQR, Interquartile Range; HF, Heart Failure; HFpEF, Heart failure with Preserved Ejection Fraction; HFrEF, Heart failure with Reduced Ejection Fraction; HRQOL, Health-Related Quality Of Life; KCCQ, Kansas City Cardiomyopathy Questionnaire; LVEF, Left Ventricular Ejection Fraction; MLHFQ, Minnesota Living with Heart Failure Questionnaire; NYHA, New York Heart Association; 6MWD, 6-Minute Walk Distance; and SD, Standard Deviation]

The initial literature search revealed 3050 articles. After exclusions, 22 studies [10 RCTs [21–30]; 12 observational [31–42]] involving 29,064 HF patients were included. Four of these studies assessed more than 1 functional health assessment tool [21, 26, 27, 38]. Majority of health assessment tools analyzed in our study focused on patients with HFrEF [n = 14], while few explored outcomes in patients with HFpEF [n = 4] and some assessed both [n = 4] (Table 1). The PRISMA flowchart (Fig. 1) summarizes that results of our literature search (Table 1) outline the baseline characteristics of all included studies. Quality assessment using the Cochrane risk of bias assessment tool for RCTs demonstrated that majority of the studies included in this meta-analysis were at a low risk of bias (Figure S1). Newcastle–Ottawa scores for observational studies indicated good methodological quality (Table S2).

**Table 1:** Study characteristics for included studies

References	HRQOL assessment tool	Proportions and/or cutoffs of HF sub-type	Geographic location	Outcomes	Number of people	Follow-up period	Age (years)	Adjusted covariates*
Cicoira et al. [41]	NYHA	90 patients (48%) had preserved systolic function	UK	Mortality	188	16 ± 10 months, range 12–41 month	CHF > 70 years old	Patient characteristics
Curtis et al. [21]	6MWD	Mean EF 34.7 (13.3 SD)	North America (USA and Canada)	All-cause mortality	541	Median 32 months	63.9 (10.8): mean (SD)	Patient characteristics, medical history, disease-related variables
				All-cause hospitalization				
Ahmed et al. [31]	NYHA	LVEF > 45% preserved	North America (USA and Canada)	All-cause mortality	988	Median of 38.5 months, with a range from 0.3 to 58.8 months	median age of 68	Medical history, disease-related variables
Friedmann et al. [24]	MLHFQ	EF ≤ 35%	USA, Canada, New Zealand	Mortality	135	27 months	60.6 (10.9): mean (SD)	Medical history, disease-related variables
Ingle et al. [32]	NYHA	LVEF = 48% (35–56) (median ± IQR)	UK	All-cause mortality	1592	Median follow-up period was 36.6 months (IQR 28.2–45.0 months)	74 median (67–80) IQR	Patient characteristics
Tate et al. [25]	MLHFQ	LVEF of ≤ 35%	North America (USA and Canada)	Mortality	2708	48 months	60 (13): mean (SD)	Patient characteristics, disease-related variables
Frankenstein et al. [33]	NYHA	LVEF ≤ 40%	Germany	All-cause mortality	1035	Mean follow-up of 52.9 ± 36.2 months	54.9 (11.5): mean (SD)	Patient characteristics(age or anthropometric variables)
Alahdab et al. [26]	NYHA + 6MWD	EF 29 ± 40%	Chicago	Mortality	198	40 months	55.7 (12.9): mean (SD)	Multivariate analyses (variables Not mentioned)
				hospitalization				
Hole et al. [34]	MLHFQ	13% of patients had an EF above 50%	Norway	Mortality	1778	60 months	Mean (73)	Patient characteristics, disease-related variables
Boxer et al. [35]	NYHA	LVEF ≤ 40%	USA	Mortality	60	4 years	78 (12): mean (SD)	Patient characteristics (age)
Pressler et al. [36]	MLHFQ	LVEF ≤ 40%	5 outpatient clinic sites in the Midwest	Mortality	166	12 months	65.6 (13.8): mean (SD)	Patient characteristics, disease-related variables, socioeconomic
Zuluaga et al. [37]	MLHFQ	At follow-up, 37.3% alive and 48.3% dead had less than 45% EF	Spain	Mortality	416	84 months	75.3 (6.1): mean (SD)	Patient characteristics, medical history, disease-related variables, socioeconomic
Manzano et al. [27]	NYHA + 6MWD	Ejection fraction ≤ 35%	Multicenter	All-cause mortality	1400	21 months	seniors ≥ 70 years	Patient characteristics, medical history, disease-related variables
Hoekstra et al. [30]	MLHFQ	33% had an LVEF > 40%	Netherland	Mortality	661	36 months	71(11): mean (SD)	Patient characteristics, medical history, disease-related variables
Ingle et al. [38]	NYHA + 6MWD	LVEF < 45%	UK	All-cause mortality	1667	5 years	age 72 [65–77]	Patient characteristics, disease-related variables for 6MWT; unadjusted for NYHA analysis
Ingle et al. [39]	6MWD	LVEF < 45%	UK	All-cause mortality	600	Median 8 years, 1 year data given	78median [72–84] IQR	Patient characteristics, medical history, disease-related variables
Pokharel et al. [28]	KCCQ	[58.2% (7.8) overall] HFpEF and HFrEF patients	Multicenter	All-cause mortality	3897	3.3 mean (1.4 years sd)	71.5 (9.7) mean (SD)	Patient characteristics, disease-related variables, medical history, devices
Luo et al. [23]	KCCQ	Reduced ejection fraction (≤ 35%)	Multicenter	All-cause mortality	2038	3 months	60 median (52–68 IQR)	Patient characteristics, medical history, disease-related variables
Bundgaard et al. [29]	MLHFQ	(LVEF) ≤ 35%	Denmark	Mortality	1116	Median 67 months (IQR 47–83)	63 years (IQR 56–71)	Patient characteristics, disease-related variables, medical history, devices
Grundtvig et al. [40]	NYHA + 6MWD	LVEF < 40% in 71%, 40–49% in 20.7%, ≥ 50% in 8.3%	Norway	All-cause mortality	5519	Median follow-up of 24 (14–36) months	68.6 (12.1): mean (SD)	Patient characteristics, disease-related variables, medical history
Matsumoto et al. [22]	6MWD	LVEF ≤ 35%	USA, Canada, Europe, and Argentina	All-cause mortality	2102	Median follow-up was 3.4 years (IQ range 2.0–5.0 years)	62.9 (11.2): mean (SD)	Patient characteristics, disease-related variables, medical history
				hospitalization				
Sepehrvand et al. [42]	KCCQ	HFpEF 59.0 (52.0, 65.3) and HFrEF 37.1 (27.8, 43.8), (median ±IQR)	Canada	Mortality	259	12 months	68.0 (57.0, 77.0)	Patient characteristics, medical history

^*^Adjusted Covariates: At least one of the following factors were included in each of the subheadings

Patient characteristics: age, sex

Medical History: additional comorbidities

Disease-related variables: estimated glomerular filtration rate (eGFR), systolic blood pressure (SBP), NYHA class, NT-proBNP, LVEF, serum markers (creatinine, uric acid), HF medication

Socioeconomic: education, social interaction, emotional support

Devices: cardiac resynchronization therapy pacemaker (CRT-P), implantable cardioverter-defibrillator (ICD)

The evaluation of methodological and conceptual quality or rigor according to the criteria of Gill and Feinstein et al. [43, 44] (Table S3) revealed that only 1 study (4%) of the 23 provided a definition of the concept QOL (criterion 1). In 15 of the papers (65%), the investigators stated the domains they measured as part of QOL (criterion 2). In 16 of the papers (70%), the investigators gave a specific reason for the choice of instrument to measure QOL (criterion 3). In 10 (44%) of the studies, the investigators had aggregated results from multiple items, domains, or instruments into a single composite score for QOL (criterion 4). In 8 studies (35%) fulfilled criterion 5, concerning whether patients were asked to give their own global rating of QOL by a single item at the end of the questionnaire. However, evaluation of the studies showed that criteria 7–10 were not fulfilled.

We investigated the association between NYHA functional class and all-cause mortality and hospitalization***.*** Of the 9 studies (n = 12,647 HF patients), 2 were exploratory analyses of RCTs [26, 27] and 7 were prospective cohorts [31–33, 35, 38, 40, 41] with a median follow-up ranging between 1 and 24 months. Four of these studies recruited patients with HFrEF [27, 33, 35, 38] only, 1 study recruited HFpEF patients [31], and 4 studies recruited patients of all subtypes [26, 32, 40, 41]. The studies typically adjusted for demographics, comorbidities and etiology of HF. Relative to NYHA I, NYHA functional classes II-IV were significant predictors of mortality.

**Fig. 2:**
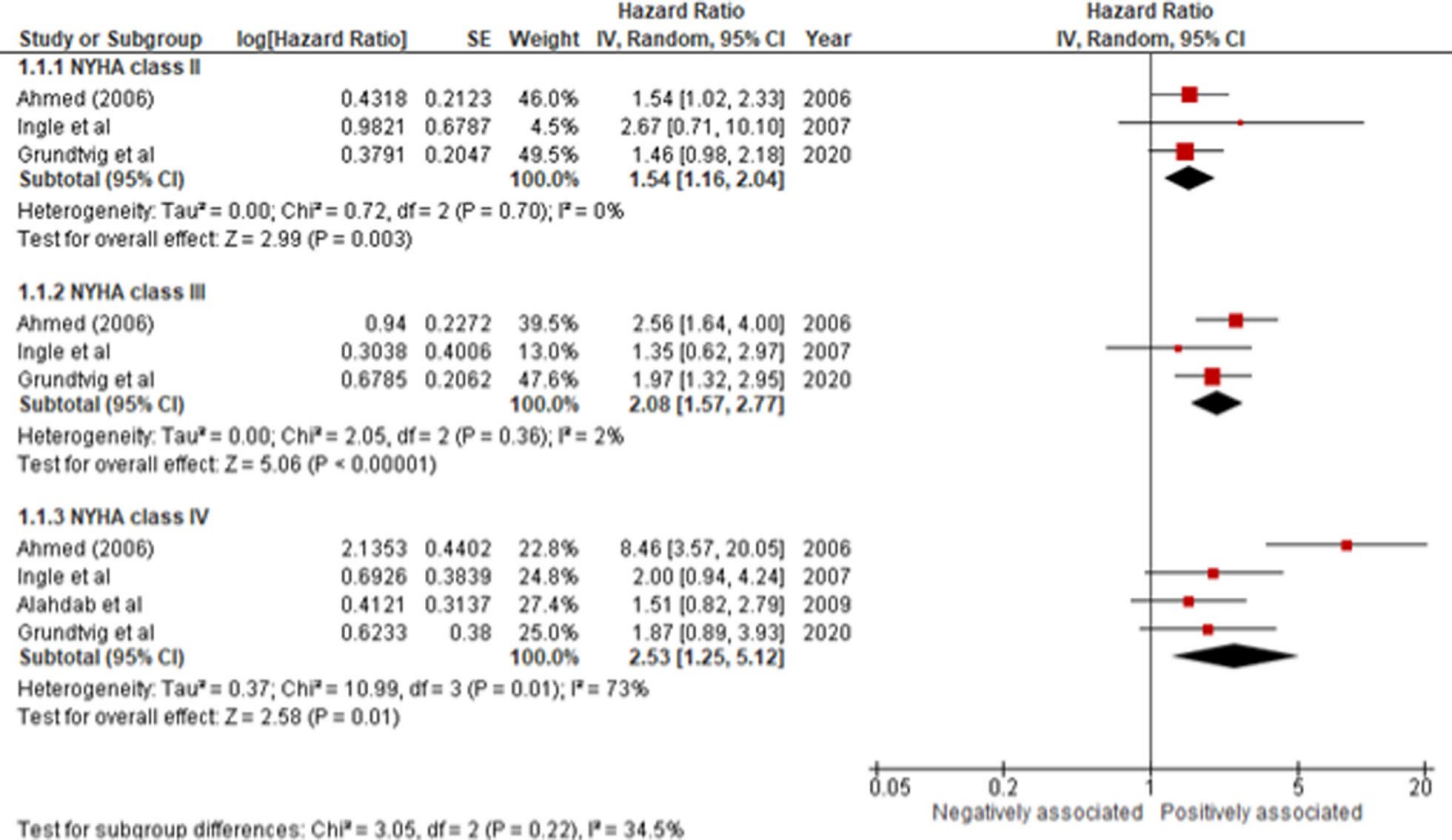
NYHA II, III, and IV compared to NYHA I, with all-cause mortality in HF patients. [CI, Confidence Interval; HR, Hazard Ratio; NYHA, New York Heart Association; and SE, Standard Error]

NYHA class II (HR 1.54 [1.16–2.04]; *I*^2^ = 0%; *p* < 0.01), NYHA class III (HR 2.08 [1.57–2.77]; *I*^2^ = 2%; *p* < 0.01), and NYHA class IV (HR 2.53 [1.25–5.12]; *I*^2^ = 73%; *p* = 0.01) were independently associated with increased risk of mortality compared with baseline NYHA functional class I (Fig. 2). Leave-one-out sensitivity analysis showed Ahmed et al. [31] to have a disproportionate effect on the heterogeneity of the results of NYHA IV. Removal of this study from NYHA class IV led to a reduction in heterogeneity while the results remained significant (HR 1.74 [1.16–2.60]; *I*^2^ = 0%; *p* < 0.01).

Subgroup analysis based on geographic regions showed NYHA classes II, III, and IV to be associated with increased mortality in both Europe and North America (Figures s2–s4). NYHA class IV had a stronger association with mortality in European regions (HR 2.70 [1.64–4.45]; *I*^2^ = 90%; *p* < 0.01) than North America (HR 1.93 [1.14–3.28]; *I*^2^ = 0%; *p* = 0.01) (Figure s4).

Studies which did not mention any baseline NYHA functional class were also pooled together to depict a per point increase, which similarly revealed an increased association with mortality (HR 1.70 (1.49–1.95]; *I*^2^ = 37%; *p* < 0.01) (Figure S5).

We also investigated the association between 6MWD-test and all-cause mortality and hospitalization***.*** Of the 7 studies (n = 12,027 HF patients), 4 were exploratory analysis of RCTs [21, 22, 26, 27] and 3 were prospective cohort studies [38–40] with a median follow-up duration between 1.5 and 5 years. Out of these, 4 of these studies recruited patients with HFrEF [22, 27, 38, 39], while the remaining three recruited patients of all subtypes. The studies typically adjusted for demographics, comorbidities, and NYHA. Three of these studies investigated mortality and hospitalization with ≤ 200 m distances covered by the participants. Of these, 2 studies were conducted in North America [21, 26], while one was multicenter [22]. The other 4 studies reported distance measured for 10 m increments for mortality in European regions [27, 38–40].

**Fig. 3:**
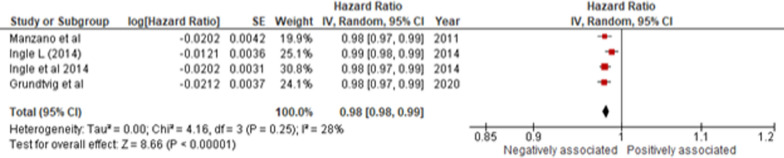
6MWD on a continuous scale at 10-m intervals with all-cause mortality in HF patients. [CI, Confidence Interval; HR, Hazard Ratio; SE, Standard Error; and 6MWD, 6-Minute Walk Distance]

Overall, in the 10 m increment studies, a significant association was observed between a lower 6MWD and mortality (HR 0.98 [0.98–0.99]; *I*^2^ = 28%; *p* < 0.01) in European regions (Fig. 3). However, of studies that dichotomized 6MWD, a distance ≤ 200 m was not significantly associated with mortality (HR 1.42 [0.86–2.32]; *I*^2^ = 76%; *p* = 0.17) (Figure S6) or hospitalization (HR 1.38 [0.91–2.07]; *I*^2^ = 84%; *p* = 0.13) (Figure S7). Upon sensitivity analysis, removal of the only study [22] that measured 6MWD using linear splines led to a reduction in heterogeneity and a significant association between ≤ 200 m 6MWD and mortality (HR 1.85 [1.22–2.80]; *I*^2^ = 0%; *p* < 0.01) and hospitalization (HR 1.69 [1.28–2.22]; *I*^2^ = 0%; *p* < 0.01) (Figures s8–s9) [22].

**Fig. 4:**
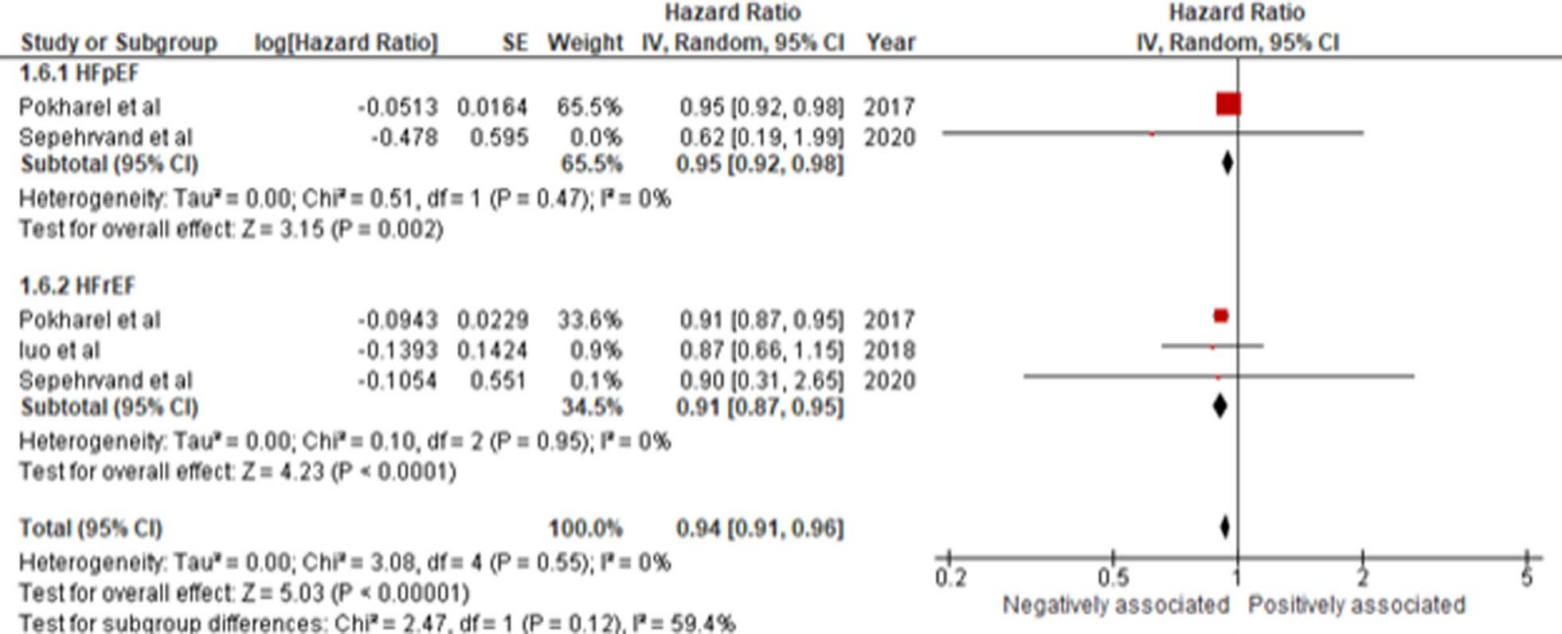
Increase of ≥ 5 point in KCCQ-OSS with all-cause mortality in HFpEF and HFrEF patients. [CI, Confidence Interval; HR, Hazard Ratio; HFpEF, Heart Failure with Preserved Ejection Fraction; HFrEF; Heart Failure with Reduced Ejection Fraction; KCCQ-OSS, Kansas City Cardiomyopathy Questionnaire; Overall Summary Score; and SE, Standard Error]

We also studied the association between KCCQ-OSS and all-cause mortality. Among 3 RCTs (n = 6,194 HF patients), 2 recruited patients with HFpEF and HFrEF [28, 42], while 1 recruited only HFrEF patients [23], with a median follow-up duration between 0.25 and 3.3 years. The RCTs typically adjusted for demographics and comorbidities. Overall, a 5-point or higher increase (≥ 5 point) in KCCQ-OSS was associated with decreased mortality (HR 0.94 [0.91–0.96]; *I*^2^ = 0%; *p* < 0.01). Upon subgroup analysis by HF subtypes, increments in KCCQ-OSS revealed a significant association with decreased mortality in both HFpEF (HR 0.95 [0.92 -0.98]; *I*^2^ = 0%; *p* < 0.01) and HFrEF (HR 0.91 [0.87–0.95]; *I*^2^ = 0%; *p* < 0.01) subgroups (Fig. 4). However, no subgroup differences in the predictive ability of KCCQ-OSS were observed when the HF subtypes were compared with each other (*p* = 0.12). Additionally, no geographical analyses could be conducted due to a lack of data.

**Fig. 5:**
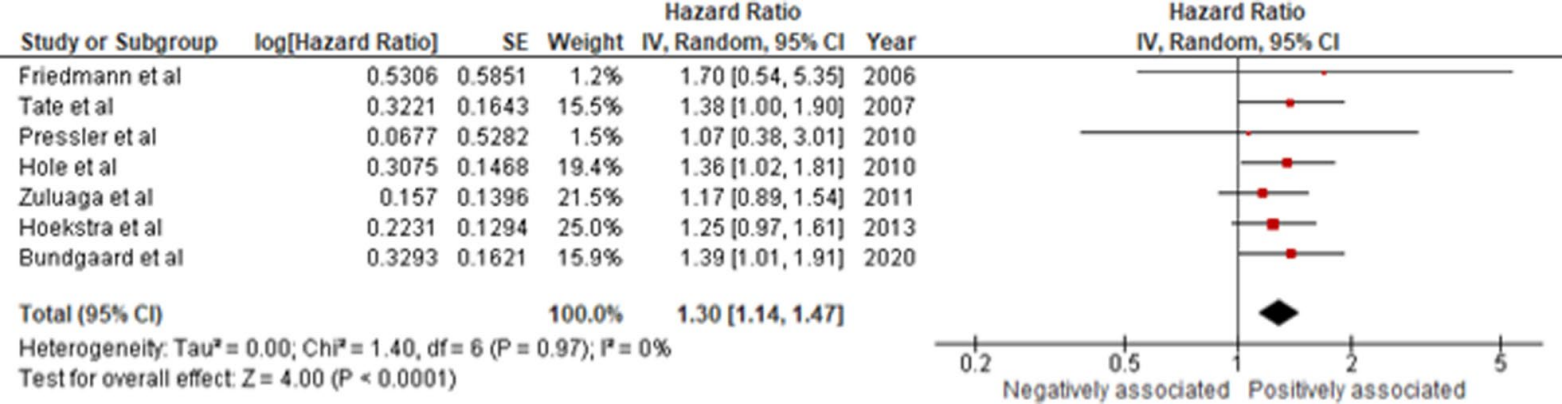
MLHFQ at a cutoff value of > 45 with all-cause mortality in HF patients. [CI, Confidence Interval; HR, Hazard Ratio; MLHFQ, Minnesota Living with Heart Failure Questionnaire; and SE, Standard Error]

Additionally, we analyzed the association between MLHFQ and all-cause mortality. Of the 7 studies (n = 6,980 HF patients), 4 were exploratory RCTs [24, 25, 29, 30] and 3 were prospective cohort studies [34, 36, 37] with a median follow-up duration between 1 and 5.5 years. Out of these, 4 of these studies recruited patients with HFrEF [24, 25, 29, 36], while the remaining 3 recruited patients of all subtypes [30, 34, 37]. The studies typically adjusted for demographics and disease-related variables such as systolic blood pressure (SBP) and NYHA. Our results revealed that a high MLHFQ score (> 45) was significantly associated with increased mortality (HR 1.30 [1.14–1.47]; *I*^2^ = 0%; *p* < 0.01) (Fig. 5). Furthermore, a subgroup analysis of geographical variation revealed a significantly higher association of MLHFQ with mortality in North America (HR 1.40 [1.03–1.91]; *I*^2^ = 0%; *p* < 0.01) compared with Europe (HR 1.25 [1.07–1.46]; *I*^2^ = 0%; *p* < 0.01) (Figure S10).

## Discussion

This meta-analysis involving 29,064 HF patients suggests that clinician-reported outcomes like NYHA and 6MWD for 10 m intervals and PROs such as KCCQ-OSS (≥ 5 points) and > 45 cutoff for MLHFQ are all significant predictors of mortality and/or hospitalization. Majority of health assessment tools analyzed in our study focused on patients with HFrEF [n = 14], while few explored outcomes in patients with HFpEF [n = 4] and some assessed both [n = 4] (Table 1). The models of the underlying studies adjusted for baseline patient characteristics and medical comorbidities.

HF often constitutes an unpredictable disease trajectory, with majority patients having a markedly impaired quality of life. The complex nature of this disease coupled with diverse pathophysiology and subsequent clinical outcomes entails the inclusion of more patient-centered outcomes which incorporates the patient’s perspective and lived experience, thereby including measures and variables which can better assess the trajectory of disease course [45]. Although assessment of mortality and hospitalization endpoints in clinical trials remains critical, the current data suggest that functional status and patient-reported outcomes offer a complementary assessment of disease burden and trajectory that carries strong prognostic value. Inclusion of these tools confers several advantages. Routine serial assessment of patient-reported outcomes and functional assessments may serve as reliable indicators of disease trajectory [46–48]. Further, compared to other invasive assessment tools such as biomarkers and implantable devices, the noninvasive nature and potential for remote assessment of health status makes it easier for both patients and clinicians to implement and routinely follow-up.

Our results revealed significant positive association of NYHA classes II, III, and IV with risk of mortality relative to NYHA class I. Only in NYHA IV did Ahmed et al. [31] contribute to significantly increased heterogeneity of the results. This could be because the study primarily focused on patients with HFpEF, while other studies mostly included patients with HFrEF. HFpEF patients tend to have various underlying etiologies as opposed to HFrEF, which is commonly associated with increased neurohormonal activation [49]. A stronger perceived association between mortality and a higher NYHA class in the study may be attributed to lack of therapeutic intervention for HFpEF patients, thereby necessitating further research into this particular populace. Additionally, our results demonstrated that classes III and IV had an increased association with mortality compared with classes I and II. These results are consistent with previous similar studies [31, 50, 51]. Patients with higher NYHA classes are usually older and have more underlying comorbidities such as kidney disease and an increased duration of cardiovascular disease which can lead to a decrease in QOL [52].

The 6MWD test, compared with the more commonly used cardiopulmonary exercise test (CPET), is a relatively affordable and easy way to assess a person’s functional health capacity [53]. Our analysis revealed that when measured on a continuous scale at 10 m intervals, the 6MWD test is especially useful in predicting mortality in patients with HFrEF. The studies pooled for 6MWD in the ≤ 200 m subgroup mainly included participants from North America with decreased LVEF, thus making this iteration more usable for HFrEF patients from the USA and Canada when predicting hospitalization. However, due to the limited number of existing studies on hospitalization from other countries, further research is needed to confirm our findings.

Similar to prior functional health assessment tools, our results demonstrated a significant association between a ≥ 5-point increase in the KCCQ-OSS score, and a decrease in mortality for both HFpEF and HFrEF. One study suggested the inclusion of biomarkers along with KCCQ-OSS to improve its ability in predicting clinical outcomes in patients with HFpEF [42]. Moreover, Huang et al. [54] demonstrated that using only the physical independence and social interaction components of the KCCQ score may also provide significantly better prognosis in HFpEF patients. Therefore, future studies should explore the accuracy of KCCQ score utility in patients with HFpEF.

Furthermore, our results assessing prognostic ability of MLHFQ scores with mortality using the standardized cutoff of > 45 concur with results of a prior meta-analysis, wherein higher MLHFQ scores indicate poor functional status, thereby increasing the risk of death. Majority studies analyzing MLHFQ score included patients with HFrEF, thus making it more suitable to determine prognosis in this cohort.

## Conclusion

In our study, majority of the functional health assessment tools were tested in North American cohorts, followed by European regions, while none of the studies reported results in rest of the world. This variation can possibly be due to the fact that most HF RCTs are conducted in North America and Europe [55]. Moreover, PRO data from HF RCTs are not commonly collected overall, but after adjusting for trial factors, are more commonly collected in trials led in Central/South America [56]. Therefore, by using readily available prognostic tools like NYHA, KCCQ, MLHFQ, and 6MWD, healthcare professionals in lower income countries may have decreased dependence on laboratory testing, such as natriuretic peptides or echocardiograms, which may not be widely available or affordable in many underdeveloped regions [12].

This meta-analysis has a few limitations that should be considered while interpreting the results. First, this meta-analysis included observational studies and secondary analyses of RCTs which are prone to residual bias. Second, differences in HF etiologies, study designs, interventions, patient, and trial characteristics present in the patient population may have contributed to clinical heterogeneity. Third, the follow-up ranges for most studies were variable, with some studies reporting longer follow-up periods. Short-term follow-ups are more useful when evaluating disease prognosis. However, long-term prognosis can overestimate progress by showing better recovery or can show worse decline in health. Additionally, when assessing ability of the tools to predict mortality, no specific time frame was mentioned in the studies. We did, however, include the range of median follow-up periods for each tool in their respective section. Lastly, the studies focusing on HF with preserved EF were few. Consequently, we were only able to make limited comparisons of clinical outcomes between HFrEF and HFpEF; hence, our results are exploratory for the HFpEF subtype.

Our findings suggest that NHYA, 6MWD (per 10 m), and MLHFQ provide significant prognostic value in predicting all-cause mortality for HF patients, particularly those with reduced EF. Further research is needed to assess prognostic impact of functional status in patients with HF in regions outside North America and Europe.

### Supplementary Information

**Additional file 1**.

## Data Availability

All data generated and/or analyzed during this study are included in this published article.

## Abbreviations

AHA: American Heart Association
CI: Confidence interval
CPET: Cardiopulmonary exercise test
EF: Ejection fraction
ESC: European Society of Cardiology
FDA: Food and Drug Administration
HF: Heart failure
HFpEF: Heart Failure with preserved Ejection Fraction
HFrEF: Heart Failure with reduced Ejection Fraction
HR: Hazard ratio
KCCQ: Kansas City Cardiomyopathy Questionnaire
KCCQ-OSS: Kansas City Cardiomyopathy Questionnaire Overall Summary Score
LVEF: Left ventricular ejection fraction
MLHFQ: Minnesota Living with Heart Failure Questionnaire
NYHA: New York Heart Association
PRISMA: Preferred Reporting Items for Systematic review and Meta-Analyses
PRO: Patient-reported outcome
QOL: Quality of life
RCT: Randomized control trial
SBP: Systolic blood pressure
SE: Standard error
6MWD: 6-Minute walk distance
