# Supplementary material for: Prognostic value of quality of life and functional status in patients with heart failure: a systematic review and meta-analysis
Source: Egypt Heart J. 2024 Aug 5;76:97. doi: 10.1186/s43044-024-00532-z (PMC11300417; doi:10.1186/s43044-024-00532-z)

**Supplementary File**

**Table S1: Detailed Search Strategy**

**Table S2: Risk of bias assessment via New-castle-Ottawa Quality Assessment Scale criteria for Non-Randomized Trials**

**Table S3: Evaluation of methodological and conceptual rigor according to the criteria from Gill and Feinstein (N = 23)**

**Figure S1: Cochrane Risk of bias Summary at Study Level for Randomized Controlled Trials**

**Figure S2: Forest plot displaying geographic variations of NYHA functional class II compared to baseline NYHA functional class I, with all-cause mortality outcomes in heart failure (HF) patients**

**Figure S3: Forest plot displaying geographic variations of NYHA functional class III compared to baseline NYHA functional class I in European regions, with all-cause mortality outcomes in heart failure (HF) patients**

**Figure S4: Forest plot displaying geographic variations of NYHA functional class IV compared to baseline NYHA functional class I, with all-cause mortality outcomes in heart failure (HF) patients**

**Figure S5: Forest plot displaying the associations of NYHA functional class (per-point -increase) with all-cause mortality outcomes in Heart Failure (HF) patients**

**Figure S6: Forest plot displaying the associations of 6MWD on a dichotomous scale equal to or below 200 meters (≤ 200m) with all-cause mortality outcomes in Heart Failure (HF) patients.**

**Figure S7: Forest plot displaying the associations of 6MWD on a dichotomous scale equal to or below 200 meters (≤ 200m), with all-cause hospitalization outcomes in Heart Failure (HF) patients.**

**Figure S8: Forest plot displaying geographic variation of 6MWD on a dichotomous scale equal to or below 200 meters (≤ 200m) in European regions, with all-cause mortality outcomes in Heart Failure (HF) patients**

**Figure S9: Forest plot displaying geographic variation of 6MWD on a dichotomous scale equal to or below 200 meters (≤ 200m) in European regions, with all- cause hospitalization outcomes in Heart Failure (HF) patients**

**Figure S10: Forest plot displaying geographic variations of MLHFQ at a cut off value of >45 with all-cause mortality outcomes in heart failure (HF) patients**

**Supplemental Table S1: Detailed Search Strategy**

| MEDLINE  3047 results | ("mortality"[MeSH Terms] OR "mortality"[All Fields] OR "mortalities"[All Fields] OR "mortality"[MeSH Subheading] OR ("death"[MeSH Terms] OR "death"[All Fields] OR "deaths"[All Fields]) OR ("rehospitalization"[All Fields] OR "rehospitalizations"[All Fields] OR "rehospitalized"[All Fields]) OR ("hospital s"[All Fields] OR "hospitalisation"[All Fields] OR "hospitalization"[MeSH Terms] OR "hospitalization"[All Fields] OR "hospitalised"[All Fields] OR "hospitalising"[All Fields] OR "hospitality"[All Fields] OR "hospitalisations"[All Fields] OR "hospitalizations"[All Fields] OR "hospitalize"[All Fields] OR "hospitalized"[All Fields] OR "hospitalizing"[All Fields] OR "hospitals"[MeSH Terms] OR "hospitals"[All Fields] OR "hospital"[All Fields])) AND ("QoL"[All Fields] OR ("qualities"[All Fields] OR "quality"[All Fields] OR "quality s"[All Fields]) OR "nyha"[All Fields] OR "kccq"[All Fields] OR "mlhfq"[All Fields] OR "6mwd"[All Fields] OR "6mwt"[All Fields]) AND ("clinical trials as topic"[MeSH Terms] OR ("clinical"[All Fields] AND "trials"[All Fields] AND "topic"[All Fields]) OR "clinical trials as topic"[All Fields] OR "trial"[All Fields] OR "trial s"[All Fields] OR "trialed"[All Fields] OR "trialing"[All Fields] OR "trials"[All Fields] OR "observational"[All Fields] OR ("observability"[All Fields] OR "observable"[All Fields] OR "observables"[All Fields] OR "observation"[MeSH Terms] OR "observation"[All Fields] OR "observe"[All Fields] OR "observed"[All Fields] OR "observer"[All Fields] OR "observer s"[All Fields] OR "observers"[All Fields] OR "observes"[All Fields] OR "observing"[All Fields] OR "watchful waiting"[MeSH Terms] OR ("watchful"[All Fields] AND "waiting"[All Fields]) OR "watchful waiting"[All Fields] OR "observations"[All Fields])) AND ("HF"[All Fields] OR "hfpef"[All Fields] OR "hfref"[All Fields] OR "heart failure"[All Fields]) AND ("prognosis"[MeSH Terms] OR "prognosis"[All Fields] OR "prognoses"[All Fields] OR ("predictor"[All Fields] OR "predictors"[All Fields])) |
| --- | --- |
| Cochranelibrary.com  1007 results | (mortality OR death OR rehospitalization OR hospitalisation) AND (QoL OR quality OR nyha OR kccq OR mlhfq OR 6mwd OR 6mwt) AND (trial OR observational OR observation) AND (HF OR hfpef OR hfref OR "heart failure") AND (prognosis OR predictor) |

**Supplemental Table S2: Risk of bias assessment via New-castle-Ottawa Quality Assessment Scale criteria for Non-Randomized Trials**

|  | Selection | | | | Comparability | Outcome | | |  |
| --- | --- | --- | --- | --- | --- | --- | --- | --- | --- |
| Author, Year | Representativeness of the exposed cohort | Selection of non-exposed cohort | Ascertainment of exposure | Demonstration that outcome was not PRESENT at start of study | Comparability of groups on the basis of analyses | Assessment of outcomes | Was follow up long enough for outcomes to occur? | Adequacy of follow up of cohort | Total score |
| Cicoira M et al (2001) | 0 | 0 | 1 | 1 | 2 | 1 | 1 | 1 | 7 |
| Ahmed et al (2006) | 1 | 0 | 1 | 1 | 2 | 1 | 1 | 0 | 7 |
| Ingle et al (2007) | 0 | 0 | 1 | 1 | 2 | 1 | 1 | 1 | 7 |
| Frankenstein L et al (2008) | 0 | 0 | 1 | 1 | 2 | 1 | 1 | 1 | 7 |
| Boxer et al (2010) | 0 | 0 | 1 | 1 | 2 | 1 | 1 | 0 | 6 |
| Hole et al (2010) | 1 | 0 | 1 | 1 | 2 | 1 | 1 | 0 | 7 |
| Pressler et al (2010) | 1 | 1 | 1 | 1 | 2 | 0 | 1 | 0 | 7 |
| Zuluaga et al (2011) | 0 | 0 | 1 | 1 | 2 | 1 | 1 | 1 | 7 |
| Ingle L (2014) | 1 | 0 | 1 | 1 | 2 | 1 | 1 | 1 | 8 |
| Ingle L et al (2014) | 1 | 1 | 1 | 1 | 2 | 1 | 1 | 1 | 8 |
| M. Grundtvig al (2020) | 1 | 0 | 1 | 1 | 2 | 1 | 1 | 1 | 8 |
| Sepehrvand et al (2020) | 1 | 1 | 1 | 1 | 1 | 1 | 1 | 0 | 7 |

**Supplemental Table S3: Evaluation of methodological and conceptual rigor according to the criteria from Gill and Feinstein (N = 23)**

| **Criteria** | **N** | **%** |
| --- | --- | --- |
| 1. Did the investigator give a definition of quality of life? | 1 | 4 |
| 2. Did the investigators state the domains they will measure as components of quality of life? | 15 | 65 |
| 3. Did the investigators give reasons for choosing the instrument they used? | 16 | 70 |
| 4. Did the investigator aggregate results from multiple items, domains or instruments into a single composite score for quality of life? | 10 | 44 |
| 5. Were patients asked to give their own global rating for quality of life? | 8 | 35 |
| 6. Was overall quality of life distinguished from health-related quality of life? | 0 | 0 |
| 7. Were the patients invited to supplement the items listed in the instruments offered by the investigators that they considered relevant for their quality of life? | 0 | 0 |
| 8. If so, were these supplemental items incorporated into the final rating? | 0 | 0 |
| 9. Were patients allowed to indicate which items were personally important to them? | 0 | 0 |
| 10. If so, were the importance ratings incorporated into the final rating? | 0 | 0 |

**Supplemental Figure S1: Cochrane Risk of bias Summary at Study Level for Randomized Controlled Trials**


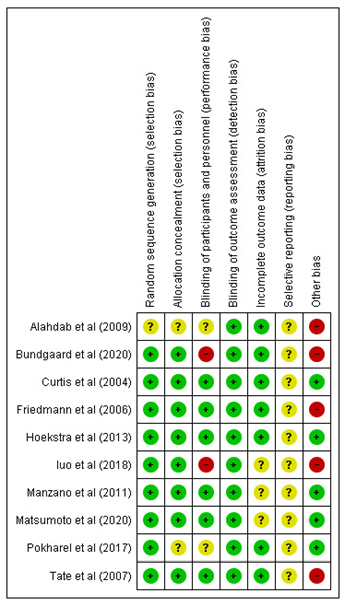


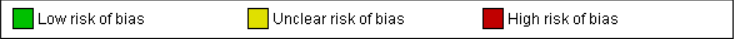


**Supplemental Figure S2: Forest plot displaying geographic variations of NYHA functional class II compared to baseline NYHA functional class I, with all-cause mortality outcomes in heart failure (HF) patients**

(CI: Confidence Interval; HR: Hazard Ratio; NYHA: New York Heart Association; SE: Standard Error)


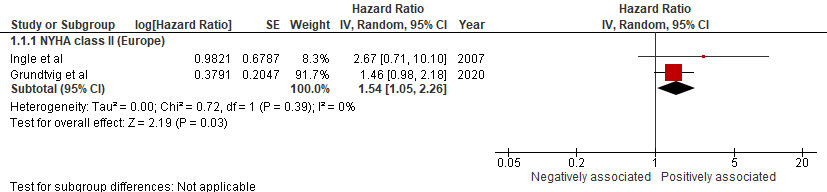


**Supplemental Figure S3: Forest plot displaying geographic variations of NYHA functional class III compared to baseline NYHA functional class I in European regions, with all-cause mortality outcomes in heart failure (HF) patients**

(CI: Confidence Interval; HR: Hazard Ratio; NYHA: New York Heart Association; SE: Standard Error)


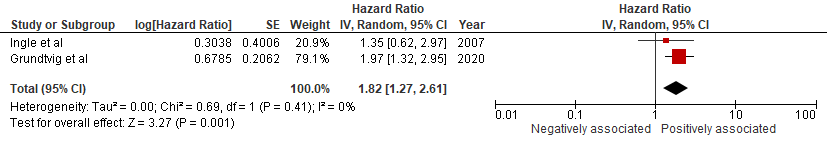


**Supplemental Figure S4: Forest plot displaying geographic variations of NYHA functional class IV compared to baseline NYHA functional class I, with all-cause mortality outcomes in heart failure (HF) patients**

(CI: Confidence Interval; HR: Hazard Ratio; NYHA: New York Heart Association; SE: Standard Error)


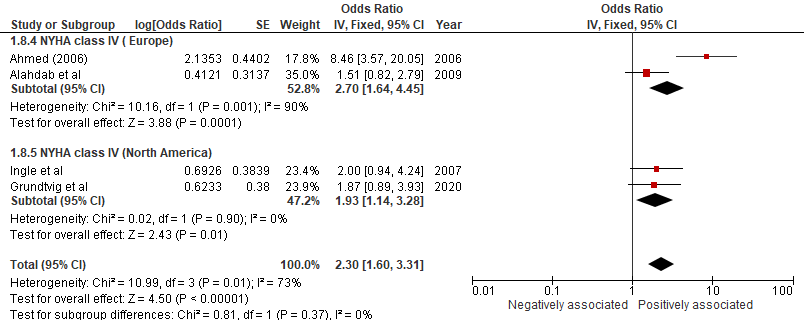


**Supplemental Figure S5:** **Forest plot displaying the associations of NYHA functional class (per-point increase) with all-cause mortality outcomes in Heart Failure (HF) patients**

(CI: Confidence Interval; HR: Hazard Ratio; NYHA: New York Heart Association; SE: Standard Error)


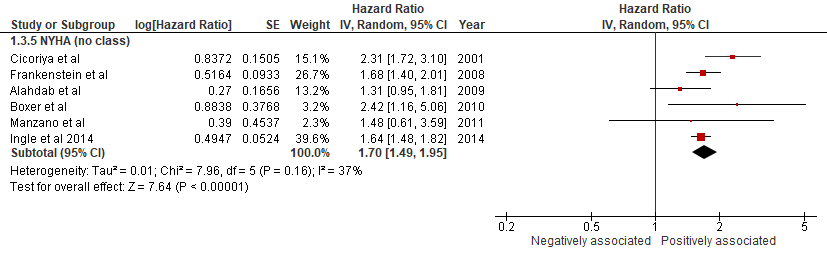


**Supplemental Figure S6: Forest plot displaying the associations of 6MWD on a dichotomous scale equal to or below 200 meters (≤ 200m) with all-cause mortality outcomes in Heart Failure (HF) patients.**

(CI: Confidence Interval; HR: Hazard Ratio; NYHA: New York Heart Association; SE: Standard Error)

**
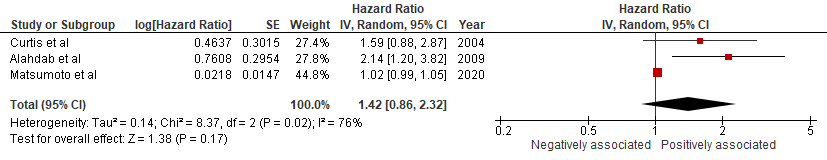
**

**Supplemental Figure S7: Forest plot displaying the associations of 6MWD on a dichotomous scale equal to or below 200 meters (≤ 200m), with all-cause hospitalization outcomes in Heart Failure (HF) patients.**

(CI: Confidence Interval; HR: Hazard Ratio; SE: Standard Error; 6MWD: 6 Minute Walk Distance)

**
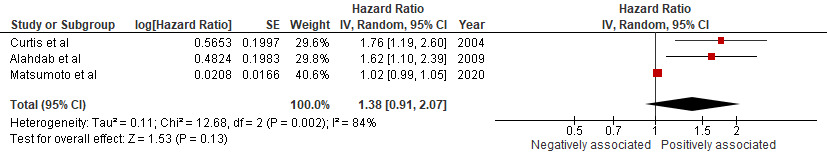
**

**Supplemental Figure S8: Forest plot displaying geographic variation of 6MWD on a dichotomous scale equal to or below 200 meters (≤ 200m) in European regions, with all- cause mortality outcomes in Heart Failure (HF) patients**

(CI: Confidence Interval; HR: Hazard Ratio; SE: Standard Error; 6MWD: 6 Minute Walk Distance)


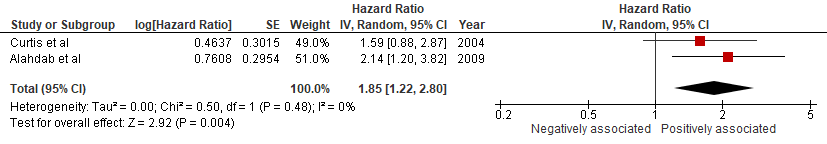


**Supplemental Figure S9: Forest plot displaying geographic variation of 6MWD on a dichotomous scale equal to or below 200 meters (≤ 200m) in European regions, with all- cause hospitalization outcomes in Heart Failure (HF) patients**

(CI: Confidence Interval; HR: Hazard Ratio; SE: Standard Error; 6MWD: 6 Minute Walk Distance)


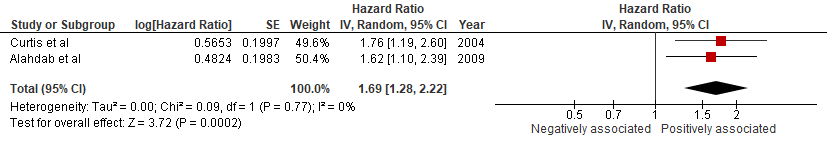


**Supplemental Figure S10: Forest plot displaying geographic variations of MLHFQ at a cut off value of >45 with all-cause mortality outcomes in heart failure (HF) patients**

(CI: Confidence Interval; HR: Hazard Ratio; MLHFQ: Minnesota Living with Heart Failure Questionnaire; SE: Standard Error)


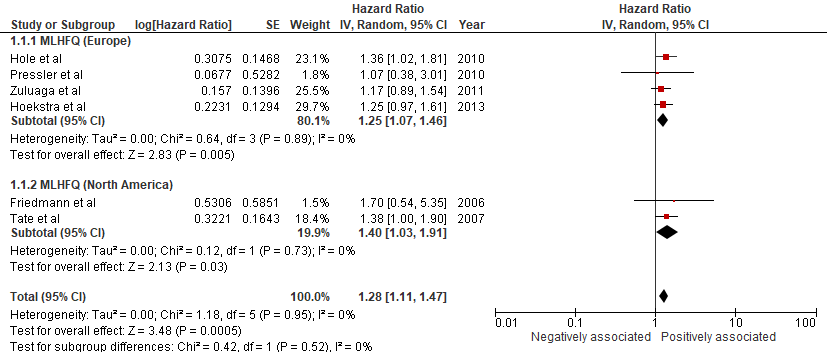

Supplement: Supplementary file 1 — Additional file 1. [file 43044_2024_532_MOESM1_ESM.docx]
